# Anti-VEGFR2 therapy delays growth of preclinical pediatric tumor models and enhances anti-tumor activity of chemotherapy

**DOI:** 10.18632/oncotarget.27148

**Published:** 2019-09-17

**Authors:** Caitlin D. Lowery, Wayne Blosser, Michele Dowless, Matthew Renschler, Lisa V. Perez, Jennifer Stephens, Bronislaw Pytowski, Heather Wasserstrom, Louis F. Stancato, Beverly Falcon

**Affiliations:** ^1^ Eli Lilly and Company, Lilly Corporate Center, Indianapolis, IN, USA

**Keywords:** angiogenesis, pediatric cancer, VEGFR2, ramucirumab, DC101

## Abstract

Vascular endothelial growth factor receptor 2 (VEGFR2) is an attractive therapeutic target in solid malignancies due to its central role in tumor angiogenesis. Ramucirumab (Cyramza^®^, LY3009806) is a human monoclonal antibody specific for VEGFR2 approved for several adult indications and currently in a phase 1 clinical trial for pediatric patients with solid tumors (NCT02564198). Here, we evaluated ramucirumab *in vitro* and the anti-murine VEGFR2 antibody DC101 *in vivo* with or without chemotherapy across a range of pediatric cancer models. Ramucirumab abrogated *in vitro* endothelial cord formation driven by cancer cell lines representing multiple pediatric histologies; this response was independent of the origin of the tumor cell-line. Several pediatric cancer mouse models responded to single agent DC101-mediated VEGFR2 inhibition with tumor growth delay. Preclinical stable disease and partial xenograft regressions were observed in mouse models of Ewing’s sarcoma, synovial sarcoma, neuroblastoma, and desmoplastic small round cell tumor treated with DC101 and cytotoxic chemotherapy. In contrast, DC101 treatment in osteosarcoma models had limited efficacy alone or in combination with chemotherapeutics. Our data indicate differential efficacy of targeting the VEGFR2 pathway in pediatric models and support the continued evaluation of VEGFR2 inhibition in combination with cytotoxic chemotherapy in multiple pediatric indications.

## INTRODUCTION

Though survival rates for pediatric cancer patients have improved dramatically since the early 1960s, cancer remains the leading cause of disease-related death in children and adolescents [1]. Following first-line therapy, approximately 25% of pediatric cancer patients will experience a relapse which generally proves fatal [2, 3]. In addition, nearly two-thirds of pediatric cancer survivors experience chronic, debilitating conditions and even secondary cancers resulting from aggressive treatment paradigms [4]. Despite dose-intensification of chemotherapy and focused efforts on understanding the molecular underpinnings of pediatric tumor types in the hopes of identifying therapeutic targets, survival rates have plateaued in recent years [5]. Therefore, identification and preclinical evaluation of novel targeted therapies in relevant pediatric model systems is necessary to support and better inform subsequent clinical investigation of these agents in pediatric indications.

The vascular endothelial growth factor (VEGF) family consists of five ligands (placental growth factor [PlGF] and VEGF-A, -B, -C, and -D) and three receptors (VEGFR1, VEGFR2, and VEGFR3) [6, 7]. Aberrant activation of VEGFR2 on endothelial cells by tumor cell-secreted VEGF-A drives angiogenesis, the development of new blood vessels from existing vessels. This new blood vessel growth supports tumor progression, local invasion, and metastasis [8, 9]. Inhibition of the VEGF pathway in cancer is thought to not only reduce the total number of vessels to support tumor growth, but also improve the function of vessels within the tumor, thereby more effectively delivering other anti-cancer therapeutics [10]. Because of the multifaceted effects on tumor vessels, the VEGF-A:VEGFR2 signaling axis has been pursued as a therapeutic target in adult solid tumors with approvals across a number of tumor histologies [11–13]. Preclinical and clinical studies have demonstrated that small molecule inhibitors of the VEGF pathway have anti-tumor activity in some pediatric malignancies; however, it is still unclear which pediatric indications may receive the most benefit from anti-VEGFR2 therapy, either alone or in combination with chemotherapy [14–18].

Ramucirumab (Cyramza^®^, LY3009806) is a fully human monoclonal antibody which specifically binds to and blocks the activation of VEGFR2 by its ligands VEGF-A, -C, and -D [19]. Currently, ramucirumab is FDA-approved for the treatment of advanced gastric or GE junction adenocarcinoma alone or in combination with paclitaxel; metastatic colorectal cancer in combination with FOLFIRI; metastatic non-small cell lung cancer in combination with docetaxel; and as a single agent in hepatocellular carcinoma patients with high alpha fetoprotein levels [20]. For pediatric patients with solid tumors, a dose-finding phase I trial for ramucirumab is currently underway (NCT02564198). Here, we tested ramucirumab or the anti-mouse VEGFR2 antibody DC101 (a ramucirumab surrogate for *in vivo* studies) in multiple pediatric cancer cell lines and xenograft mouse models with the goal of identifying specific pediatric indications that may respond to ramucirumab-mediated VEGFR2 inhibition.

## RESULTS

### Pediatric cancer cell lines produce ligands for VEGFR2

**Figure 1:**
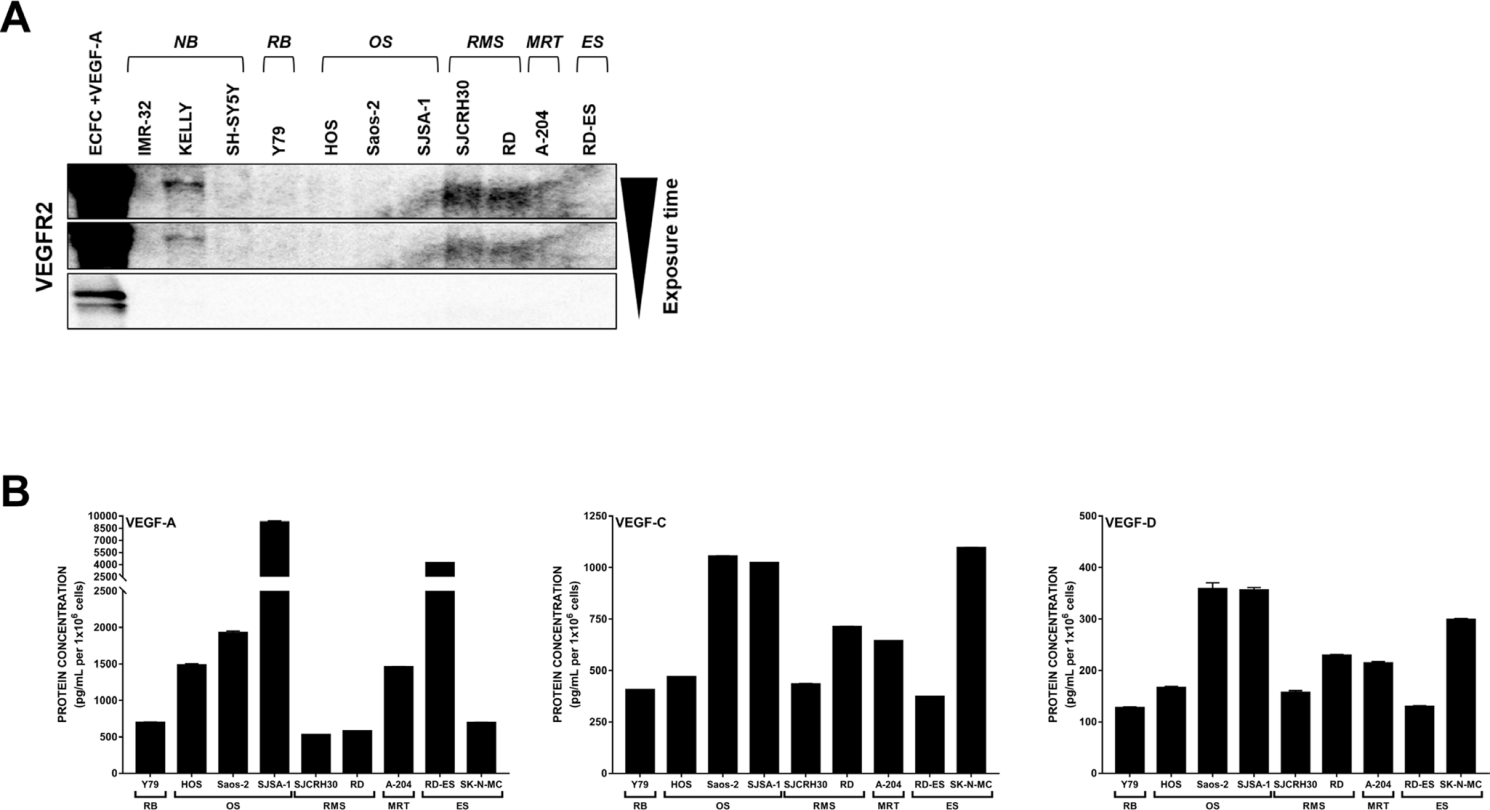
Expression of VEGFR2 and associated VEGFs are detected in pediatric cancer cell lines. (**A**) Eleven pediatric cancer cell lines were evaluated for endogenous VEGFR2 protein expression. (**B**) Endogenous levels of VEGF-A (left), -C (middle), and -D (right) protein produced by 9 pediatric cancer cell lines in co-culture conditions were assayed by ELISA. Error bars represent SEM. Note the bro ken y-axis for VEGF-A. Abbreviations: ECFC, endothelial colony forming cell; NB, neuroblastoma; RB, retinoblastoma; OS, osteosarcoma; RMS, rhabdomyosarcoma; MRT, malignant rhabdoid tumor; ES, Ewing’s sarcoma.

To first establish the expression patterns of VEGFR2 and its associated ligands in our pediatric cancer models, we profiled a panel of 11 pediatric cancer cell lines representing neuroblastoma (IMR-32, KELLY, SH-SY5Y), retinoblastoma (Y79), osteosarcoma (HOS, Saos-2, SJSA-1), rhabdomyosarcoma (SJCRH30 [alveolar RMS], RD [embryonal RMS]), malignant rhabdoid tumor (A-204), and Ewing’s sarcoma (RD-ES) for VEGFR2 protein expression (Figure 1A). As expected, VEGFR2 was absent from the majority of cancer cell lines and detected in only 3 out of the 11 cell lines (KELLY, SJCRH30, and RD) at much lower levels than the VEGF-A-stimulated endothelial colony forming cell (ECFC) control.

Tumor cells can activate VEGFR2 on endothelial cells (and thus promote neovascularization) through production and secretion of VEGF-A, -C, and -D. We previously determined that the neuroblastoma cell lines (IMR-32, KELLY, and SH-SY5Y) produce VEGF-A, -C, and -D in co-culture conditions [21]. We determined that these ligands were also present in media collected from each of the 9 additional non-neuroblastoma cell lines grown in co-culture conditions (Figure 1B). VEGF-A production was the most varied, from over 9000 pg/mL detected in SJSA-1 osteosarcoma media to approximately 525 pg/mL in the SJCRH30 alveolar RMS media. Conversely, VEGF-C levels were generally below 1000 pg/mL and VEGF-D was more uniformly expressed at concentrations below 400 pg/mL across all cell lines tested.

### Ramucirumab impedes both VEGF- and tumor-driven cord formation

**Figure 2:**
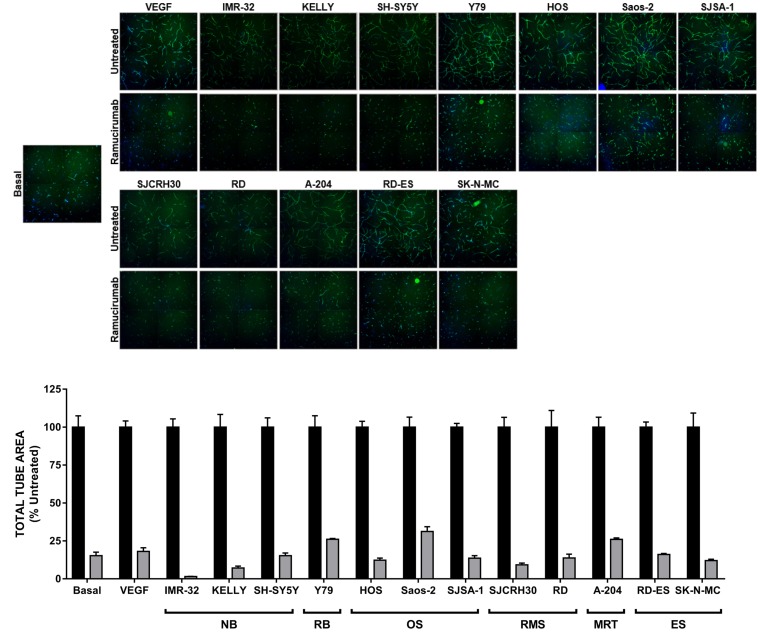
Ramucirumab blocks both VEGF-A- and pediatric tumor cell-driven cord formation *in vitro*. *(Top)* Representative images of cords from each condition are shown. *(Bottom)* Total tube area of VEGF-A- and tumor cell-driven cords is presented, with data for each cell line normalized to its respective untreated control. Black bars: untreated; gray bars: treated with 10 μg/mL ramucirumab. Error bars represent SEM.

We next tested the ability of pediatric cancer cell lines to support *in vitro* endothelial cord formation [22]. As adipocyte derived stem cells (ADSCs) and ECFCs grown together in co-culture conditions were shown to produce a minimal amount of VEGF-A (approximately 40 pg/mL) [22], exogenous VEGF-A was used to drive cord formation in tumor cell-free wells. Indeed, the proangiogenic factors secreted by the panel of pediatric cancer cell lines could promote the formation of cords comparable to those achieved in VEGF-A-driven assays (Figure 2). Inhibition of VEGFR2 using the monoclonal antibody ramucirumab (Cyramza^®^, LY3009806) significantly blunted cord formation promoted by either tumor cell lines or VEGF-A, as measured by a significant reduction in total tube area (≥65%) with treatment compared to controls (Figure 2). Ramucirumab-mediated reduction in tumor-driven cord formation was not a result of cancer cell death (Supplementary Figure 1), consistent with the lack of target expression in the majority of tumor cell lines tested.

### DC101 is active as monotherapy or in combination with chemotherapy in a subset of pediatric bone and soft tissue tumor models

As ramucirumab does not cross react with mouse VEGFR2, the rat anti-mouse VEGFR2 antibody DC101 [23] was used to treat 8 cell line-derived (CDX) and 21 patient-derived (PDX) xenograft mouse models representing 10 extracranial pediatric solid tumor types (Table 1 and Supplementary Table 1). Animals were treated with 20 mg/kg DC101 twice weekly for up to 4 weeks, either as a single agent or in combination with chemotherapies typically used for pediatric cancer patients.

**Table 1:** Summary of *in vivo* pediatric tumor model studies

Model	Xenograft type	Tumor type	Analysis day	Chemotherapy		DC101	Chemo	Combination	Combination effect^
				Agent	Dose, Schedule	% ΔT/C or regression on analysis day			
SJCRH30	CDX	alveolar RMS	38	Doxorubicin	5 mg/kg, Q7Dx4	**26**	**57**	**15^†‡^**	**Additive**
RD	CDX	embryonal RMS	64	Doxorubicin	5 mg/kg, Q7Dx4	**40**	**20**	**12^†^**	Less than additive
CTG-1213	PDX	embryonal RMS	26	Actinomycin D	0.25 mg/kg, D0, 21	46	62	**27^‡^**	Unknown
CTG-0926	PDX	DSRCT	28	Doxorubicin	5 mg/kg, Q7Dx4	60	**42**	**22^†‡^**	Unknown
CTG-1458	PDX	DSRCT	35	Cyclophosphamide	100 mg/kg, Q7Dx4	**50**	**42**	**8^†‡^**	**Additive**
RD-ES	CDX	Ewing’s sarcoma	28	Doxorubicin	5 mg/kg, Q7Dx4	69	**28**	**-30^†‡^**	**Additive**
CTG-0142	PDX	Ewing’s sarcoma	28	Doxorubicin	5 mg/kg, Q7Dx4	**44**	**38**	**11^†‡^**	Unknown
CTG-0785	PDX	Ewing’s sarcoma	18	Doxorubicin	5 mg/kg, Q7Dx4	**37**	**46**	**41**	Less than additive
CTG-0816	PDX	Ewing’s sarcoma	35	Doxorubicin	5 mg/kg, Q7Dx4	**3**	**3**	**-49^†‡^**	Less than additive
CTG-0994	PDX	Ewing’s sarcoma	21	Doxorubicin	5 mg/kg, Q7Dx4	**19**	78	**42^‡^**	Less than additive
CTG-1072	PDX	hepatoblastoma	17	Cisplatin	5 mg/kg, Q7Dx4	55	**55**	**52**	No effect
A-204	CDX	malignant rhabdoid tumor	52	Doxorubicin	5 mg/kg, Q7Dx4	62	**33**	**31**	No effect
IMR-32	CDX	neuroblastoma	70	Doxorubicin	3 mg/kg, Q7Dx4	**47**	**30**	**16^†‡^**	**Additive**
KELLY	CDX	neuroblastoma	49	Doxorubicin	2 mg/kg, Q7Dx3	**46**	98	**48^‡^**	No effect
SH-SY5Y	CDX	neuroblastoma	52	Doxorubicin	5 mg/kg, Q7Dx4	25	**10**	**-28**	No effect
CTG-0241	PDX	osteosarcoma	28	Doxorubicin	5 mg/kg, Q7Dx4	**51**	109	78	No effect
CTG-0242	PDX	osteosarcoma	28	Doxorubicin	5 mg/kg, Q7Dx4	56	**9**	**21**	No effect
CTG-0243	PDX	osteosarcoma	16	Doxorubicin	5 mg/kg, Q7Dx4	229	**219**	95^†‡^	No effect
CTG-1064	PDX	osteosarcoma	27	Doxorubicin	5 mg/kg, Q7Dx4	53	157	41^‡^	No effect
Y79	CDX	retinoblastoma	44	Doxorubicin	5 mg/kg, Q7Dx4	**16**	**18**	**-11**	Unknown
CTG-0331	PDX	synovial sarcoma	27	Doxorubicin	5 mg/kg, Q7Dx4	**22**	**36**	**7^‡^**	No effect
CTG-1173	PDX	synovial sarcoma	52	Gemcitabine Docetaxel	60 mg/kg, Q7Dx4 6 mg/kg, Q7Dx4	79	**-43**	**-64^†‡^**	**Additive**
CTG-1094	PDX	undifferentiated sarcoma	17	Doxorubicin	5 mg/kg, Q7Dx4	**45**	**44**	53	No effect

***p* < 0.05 compared to control**

^BLISS independence method

^†^ *p* < 0.05 compared to DC101 alone

^‡^ *p* < 0.05 compared to chemotherapy alone

**Figure 3:**
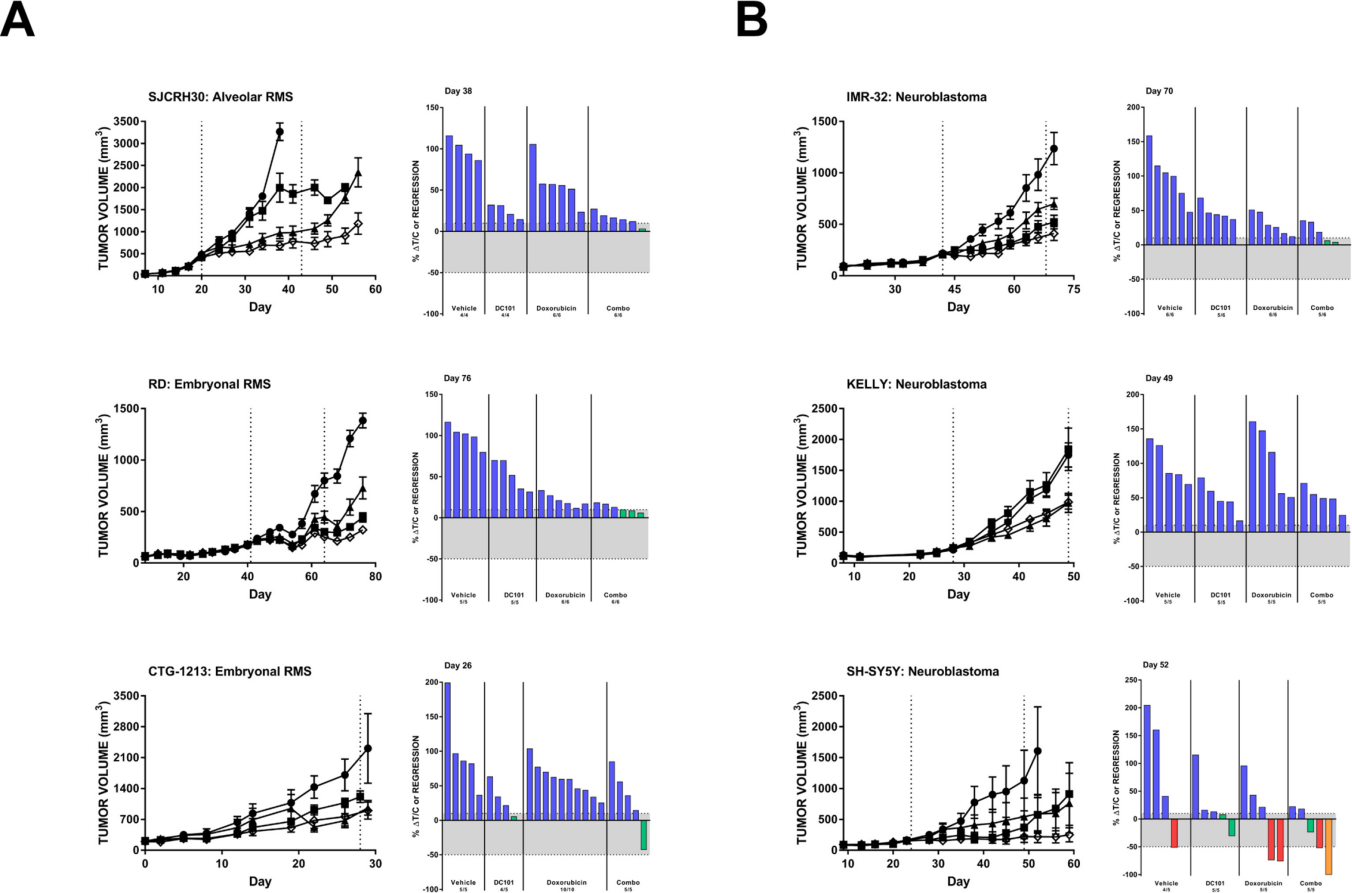
DC101 monotherapy delays tumor growth in pediatric mouse models of rhabdomyosarcoma (**A**) and (**B**) neuroblastoma. All animals were treated with control (●), DC101 (▲), doxorubicin (■), or the combination (◇). Animals were treated when tumors reached approximately 200 mm^3^. The treatment interval is represented by dotted vertical lines except where treatment began at Day 0 in which case the end of treatment is indicated by a single dotted line at Day 28. Error bars represent SEM. Waterfall plots were generated on the day indicated in the top left corner of the image (generally, the last day the majority of vehicle animals were still evaluable). Blue bars: progressive disease (PD; ≥10% ΔT/C). Green bars: stable disease (SD; <10% ΔT/C and <50% regression). Red bars: partial regression (PR; ≥50% regression and tumor volume ≥ 14 mm^3^). Orange bars: complete regression (CR; tumor volume < 14 mm^3^). RMS: rhabdomyosarcoma.

We observed that single agent DC101 significantly reduced tumor volumes compared to untreated animals in 13 of the 29 (45%) evaluated pediatric cancer models, though the responses were generally limited to tumor growth delay resulting in lower average tumor volume but not stable disease or tumor regression (Table 1). Three RMS models, one alveolar (SJCRH30) and two embryonal (RD and CTG-1213), responded to chemotherapy and DC101 as single agents. When the two drugs were combined, a significantly greater reduction in tumor volume was observed and some individual animals achieved stable disease (Figure 3A, Table 1). Similarly, all three *in vivo* mouse models of neuroblastoma were sensitive to DC101 monotherapy (Figure 3B, Table 1). While the reduction in KELLY xenograft volumes in the combination treatment arm could be attributed to DC101 alone (limited effects of doxorubicin single agent was observed in this model), improved responses with combination treatment were noted in the other two neuroblastoma models. Individual animals bearing IMR-32 or SH-SY5Y xenografts achieved stable disease or partial response and one animal with an SH-SY5Y tumor demonstrated a complete response when treated with DC101 and doxorubicin.

**Figure 4:**
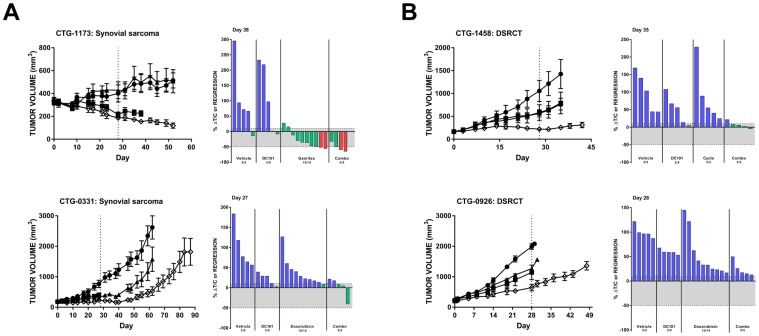
DC101 enhances the effects of chemotherapy in preclinical models of (**A**) synovial sarcoma and (**B**) DSRCT. All animals were treated with control (●), DC101 (▲), chemotherapy (■; noted in waterfall plot), or the combination (◇). Treatment began at Day 0 and ended at Day 28 (dotted vertical line). For each panel, tumor growth curves are shown on the left and waterfall plots on the right. Error bars represent SEM. Waterfall plots were generated on the day indicated in the top left corner of the image (generally, the last day the majority of vehicle animals were still evaluable). Blue bars: progressive disease (PD; ≥10% ΔT/C). Green bars: stable disease (SD; <10% ΔT/C and <50% regression). Red bars: partial regression (PR; ≥50% regression and tumor volume ≥ 14 mm^3^). Abbreviations: DSRCT, desmoplastic small round cell tumor; Gem/tax, gemcitabine plus docetaxel.

The synovial sarcoma PDX model CTG-1173 was largely nonresponsive to DC101 monotherapy; however, 8/10 animals had tumor regression following gemcitabine/docetaxel treatment. Remarkably, this response was significantly enhanced with the addition of DC101 to gemcitabine/docetaxel (Figure 4A, top; Table 1). Another synovial sarcoma PDX model (CTG-0331) responded to both single agent DC101 and doxorubicin with a tumor growth delay (Figure 4A, bottom). The combination treatment resulted in a pronounced tumor growth delay which was sustained for approximately 3 weeks following the end of treatment. Both desmoplastic small round cell tumor (DSCRT) PDX models responded to single agent treatment with either DC101 or chemotherapy (either cyclophosphamide or doxorubicin) (Figure 4B). Combination treatment of DC101 and cyclophosphamide resulted in stable disease in animals bearing CTG-1458 tumors and DC101 also enhanced the anti-tumor activity of doxorubicin in the CTG-0926 PDX model.

**Figure 5:**
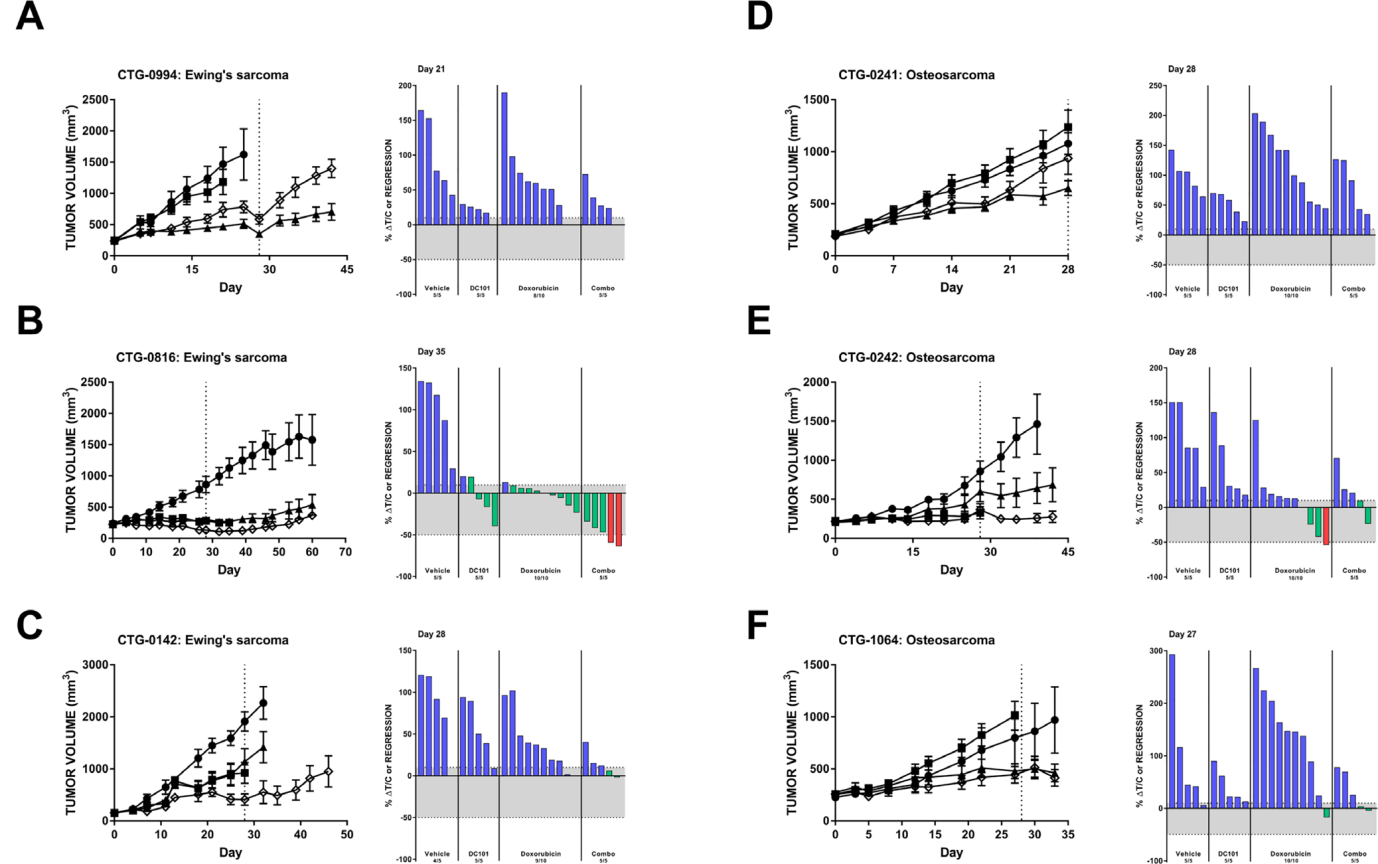
Preclinical models of Ewing’s sarcoma and osteosarcoma respond to DC101 alone and in combination with cytotoxic chemotherapy. All animals were treated with control (●), DC101 (▲), doxorubicin (■), or the combination (◇). Treatment began at Day 0 and ended at Day 28 (dotted vertical line). For each panel, tumor growth curves are shown on the left and waterfall plots on the right. Error bars represent SEM. Waterfall plots were generated on the day indicated in the top left corner of the image (generally, the last day the majority of vehicle animals were still evaluable). Blue bars: progressive disease (PD; ≥10% ΔT/C). Green bars: stable disease (SD; <10% ΔT/C and <50% regression). Red bars: partial regression (PR; ≥50% regression and tumor volume ≥ 14 mm^3^).

Several Ewing’s sarcoma models responded to *in vivo* VEGFR2 inhibition (Tables 1 and 2). Significant single agent activity of DC101 was observed in CTG-0994, which was not further enhanced with the addition of doxorubicin (Figure 5A). Stable disease was observed in the CTG-0816 Ewing’s sarcoma PDX model following DC101 monotherapy and combination with doxorubicin resulted in two animals achieving a partial response (>50% regression) (Figure 5B). The combination of DC101 and doxorubicin was superior to either single agent in animals with CTG-0142 Ewing’s sarcoma xenografts (Figure 5C). Six additional PDX models of Ewing’s sarcoma were evaluated in an ‘n of 1’ design (Table 2). One model, CTG-1651, responded to both single agent DC101 and the combination of DC101 with doxorubicin with stable disease (8.4% ΔT/C and 12% regression, respectively). The CTG-1663 model responded to DC101 monotherapy, but this response was not enhanced with the addition of doxorubicin. Three models (CTG-2003, -2113, and -2174) were most sensitive to single agent doxorubicin treatment, while CTG-0143 did not respond to either DC101 or doxorubicin monotherapy and only demonstrated a slight reduction in tumor volume with the combination.

**Table 2:** Summary of Ewing’s sarcoma ‘n of 1’ study results

Model	DC101	Doxorubicin	Combination
	% ΔT/C or regression^*^		
CTG-0143	153	176	66.4
CTG-1651	8.4	57.4	−12.1
CTG-1663	29.2	44	20.7
CTG-2003	101	42.8	103
CTG-2113	54.6	12.3	39.6
CTG-2174	54.5	3.1	52.1

^*^at end of treatment (Day 28) or date of sacrifice if prior to end of treatment.

Additional pediatric tumor models were also interrogated for their response to DC101 with or without chemotherapy (Table 1). Only 1 out of 4 osteosarcoma models (CTG-0241) had a significant reduction in tumor growth with DC101 treatment alone (Figure 5D). Two additional osteosarcoma models demonstrated some reduction in tumor growth with DC101 monotherapy, but these responses were not statistically significant (Figure 5E and 5F). Combination treatment of DC101 and doxorubicin in these models did not significantly reduce tumor growth compared to vehicle and doxorubicin alone. A-204 malignant rhabdoid tumor, Y79 retinoblastoma, CTG-1072 hepatoblastoma, and CTG-1094 undifferentiated sarcoma models responded to DC101 and/or chemotherapy alone, but the combination did not improve response (Table 1).

### Two preclinical mouse models respond to DC101 following initial treatment with doxorubicin

We explored the potential for DC101 to act as a maintenance therapy in a subset of PDX models of several histologies (Supplementary Table 1). Following 4 weeks of doxorubicin treatment, animals were split into two groups—one receiving no treatment and one receiving DC101 monotherapy. Two out of 14 models assessed responded to subsequent treatment with DC101 (Supplementary Figure 2). Pronounced inhibition of tumor growth was observed in the CTG-0331 synovial sarcoma model and tumor growth delay was noted in the CTG-0994 Ewing’s sarcoma model when DC101 was administered following completion of the doxorubicin treatment interval.

Collectively, our *in vivo* data demonstrate that DC101-mediated VEGFR2 inhibition was sufficient to delay tumor growth in alveolar and embryonal RMS, DSRCT, Ewing’s sarcoma, synovial sarcoma, and neuroblastoma. DC101 monotherapy enhanced the duration of tumor growth delay following an initial response to doxorubicin in one model of each synovial sarcoma and Ewing’s sarcoma. We also observed that the concurrent administration of DC101 and chemotherapy improved the anti-tumor response compared to control and chemotherapy alone in mice bearing models of alveolar RMS, embryonal RMS, DSRCT, Ewing’s sarcoma, neuroblastoma, and synovial sarcoma, but not osteosarcoma, hepatoblastoma, rhabdoid, or retinoblastoma.

## DISCUSSION

The low incidence of molecular aberrations combined with the relative rarity of pediatric cancer and associated preclinical models often impedes the development and clinical evaluation of novel therapies specifically for pediatric indications [5, 24, 25]. Targeting the tumor microenvironment is a potential avenue to improve patient response to existing regimens consisting of chemotherapy and/or targeted agents with unique mechanisms-of-action. As VEGFR2 is a crucial mediator of tumor angiogenesis and is predominately expressed on the endothelium rather than tumors, it is a promising therapeutic target across different cancer types. Novel agents which target tumor angiogenesis have been assessed clinically across adult and pediatric malignancies, some of which are now FDA-approved for adult indications [12, 26, 27]. However, small molecule inhibitors may block multiple kinases, leading to off-target effects and associated toxicity [28] and to date no VEGFR2 pathway inhibitor has been approved for pediatric indications. In order to identify potential pediatric indications that may benefit from specific VEGFR2 inhibition, we tested ramucirumab or the anti-murine VEGFR2 antibody DC101 in multiple preclinical models of pediatric solid tumors. We demonstrated that ramucirumab abrogated both VEGF-A- and pediatric cancer cell-driven endothelial cord formation *in vitro*; furthermore, treatment with DC101 improved response to chemotherapy in several xenograft mouse models representing a range of pediatric tumor types with high unmet need.

VEGFR2, the target of ramucirumab and DC101, is primarily expressed on endothelial cells and is largely activated by tumor cell-secreted VEGF, spurring the onset of neovascularization. [29]. Indeed, we observed that the health of pediatric tumor cell lines was not affected by *in vitro* ramucirumab treatment despite the universal reduction in cord formation across all cell lines tested. This demonstrates the need for combination treatment of VEGFR2 pathway inhibition with a cytotoxic agent to hit both the tumor and the pathogenic tumor microenvironment. We did not expect DC101 alone to promote xenograft regression in our CDX models of pediatric cancer as it only targets the mouse receptor and considered stable disease or tumor growth delay to be indicative of DC101 monotherapy activity. Indeed, multiple pediatric tumor models met these criteria, including alveolar and embryonal RMS, DSRCT, Ewing’s sarcoma, neuroblastoma, and synovial sarcoma and tumor regression was only observed when DC101 was combined with doxorubicin or another cytotoxic agent. Therefore, VEGFR2 inhibition via ramucirumab treatment may provide additional clinical benefit when added to other cytotoxic agents, including those used during pediatric cancer treatment such as vincristine, irinotecan, and temozolomide [5] or targeted agents with unique mechanisms-of-action currently being explored in the pediatric population. Ramucirumab and DC101 were active across multiple pediatric histologies, thus demonstrating the relatively tumor type-agnostic activity of VEGFR2 inhibition. However, there were some pediatric indications such as osteosarcoma that appeared less sensitive to these combinations. The best combination partners for ramucirumab may vary across tumor types and between patients. In addition, tumors can employ one of multiple intrinsic or acquired escape mechanisms to circumvent the anti-tumor activity of VEGF pathway inhibitors [30–32]; however, detailing which of these mechanisms applies to each pediatric tumor model was beyond the scope of this study. Therefore, the efficacy of potential combinations as well as the identification of ramucirumab resistance would be best explored in a clinical setting.

To our knowledge, this preclinical study is the first exploring ramucirumab and DC101 in pediatric cancer models. Our data demonstrate that VEGFR2 inhibition, either as monotherapy or in combination with chemotherapy, promotes anti-tumor responses in several pediatric cancer models and further supports clinical investigation of ramucirumab in combination with other therapies for pediatric cancer patients.

## METHODS

### Cell culture conditions

All cell lines were purchased from the American Type Culture Collection (ATCC) or DSMZ and grown in the media recommended by the respective vendor. Cell lines were maintained at 37° C in 5% CO_2_.

### Test compound

Ramucirumab (LY3009806, Eli Lilly and Company) was diluted in PBS at a stock concentration of 8.9 mg/mL. The anti-murine VEGFR2 antibody DC101 (LY3180389, Eli Lilly and Company) was suspended in phosphate-buffered saline (PBS) for *in vivo* administration.

### Western blot analysis

Cell lysis, SDS-PAGE, and immunoblotting were performed as previously described [21]. Cells pieces were lysed in 1% SDS (Fisher BioReagents, cat#BP2436-200) supplemented with 1x HALT protease and phosphatase inhibitor (ThermoFisher, cat#78441). The following antibodies were purchased from Cell Signaling Technology: VEGFR2 (cat#2479), VEGFR2 Y1175 (cat#2478), ERK1/2 (cat#4695), and ERK1/2 T202/Y204 (cat#4370). For protein assessment following ligand stimulation, cells were incubated with 10 ng/mL VEGF-A (R&D Systems cat#293VE) for 10 minutes prior to harvest and lysate generation.

### Quantification of ligand production by cancer cell lines

Pediatric cancer cell lines were grown in adipocyte derived stem cell (ADSC) conditioned media, which are the co-culture conditions for cord formation. Conditioned media was collected at the 48 hour timepoint and analyzed using the MSD Multi-Spot Assay System Angiogenesis Panel 1 (MSD, cat#K15190D) per the manufacturers’ instructions.

### *In vitro* cord formation assays

Cord formation assays were performed as previously described [21, 22, 33]. Ramucirumab was used at a final concentration of 10 μg/mL. VEGF-A (Invitrogen, cat#PHC9394) was used at 10 ng/mL in wells without tumor cells to serve as a positive control for cord formation, as a previous study demonstrated that ADSCs and endothelial colony forming cells grown in co-culture conditions produce approximately 40–50 pg/mL VEGF-A and thus need exogenous ligand for cord formation [22]. In addition, the amount of VEGF-A contributed by ADSCs and ECFCs was assumed constant across all conditions tested.

Cell viability was assessed following completion of the cord formation assay using the CellTiter Glo™ (CTG) Luminescent Cell Viability Assay (Promega, cat# G7571). Transwells containing tumor cells were removed and placed into a white, opaque bottom 96-well plate. Approximate volume was determined by measuring with a pipette, the appropriate volume of CTG reagent was added, and the CTG reagent/lysate solution was transferred to the white plate and read on a SpectraMax plate reader. For each cell line, luminescence was normalized to the average of the DMSO-treated control.

### *In vivo* evaluation of DC101

Experiments involving animals were performed in accordance with American Association for Laboratory Animal Care institutional guidelines. *In vivo* studies using cell line-derived xenograft (CDX) models were approved by the Eli Lilly and Company Animal Care and Use Committee. *In vivo* experiments utilizing patient-derived xenograft (PDX) models designated by codes starting with ‘CTG’ were conducted at Champions Oncology (Hackensack, NJ, USA).

To evaluate DC101 and chemotherapy effects on CDX growth, cells were harvested during log phase growth and resuspended in Hank’s Balanced Salt Solution (HBSS). Suspended cells were diluted 1:1 with BD Matrigel Matrix (cat#356234; RD-ES, KELLY, IMR-32, SH-SY5Y) and 5 × 10^6^ cells or 10 × 10^6^ cells (RD-ES and RD) were injected subcutaneously into the right flank of female athymic nude mice. When tumor volumes averaged ~200 mm^3^, mice were randomized into treatment groups. Animals were given vehicle (20% Captisol™ in water, pH 4), DC101, chemotherapy (doxorubicin, cisplatin, cyclophosphamide, or gemcitabine + docetaxel), or a combination of DC101 and chemotherapy. Combination partners were dependent on the tumor model. DC101 (20 mg/kg, ip) was administered twice weekly for up to 4 weeks. Chemotherapy was given for 4 weeks at the following dose and schedules: doxorubicin, 5 mg/kg (unless otherwise noted) once weekly (Q7D; iv); cisplatin, 4 mg/kg once weekly (Q7D; ip); cyclophosphamide, 100 mg/kg once weekly (Q7D; ip); gemcitabine, 60 mg/kg once weekly (Q7D; ip) + docetaxel, 6 mg/kg once weekly (Q7D; ip). For DC101 maintenance therapy-related studies, animals received doxorubicin for 4 weeks. The doxorubicin group was then split into two equal groups: one received DC101 monotherapy at the same dose/schedule as described above, the other received vehicle control. Studies were terminated when the tumors from the “doxorubicin followed by vehicle” group reached a size which necessitated euthanasia. Additional details, including specific mouse strains and number of animals per arm, are located in Supplementary Table 1.

Tumor volume was transformed to a log scale to equalize variance across time and treatment groups. Log volume data was analyzed with a two-way repeated measures analysis of variance by time and treatment using the MIXED procedures in SAS software (Version 9.3). The correlation model for the repeated measures was Spatial Power. Treated groups were compared to the control group at each time point. The MIXED procedure was also used separately for each treatment group to calculate adjusted means and standard errors at each time point. Combinations were defined as additive if the combination arm was statistically different from both of the single agent arms using the BLISS independence method. Waterfall plots were generated on the day prior to splitting of treatment groups or the last day the majority of vehicle animals were able to be evaluated prior to sacrifice.

## SUPPLEMENTARY MATERIALS



## Abbreviations

VEGFR1: vascular endothelial growth factor receptor 1
VEGFR2: vascular endothelial growth factor receptor 2
VEGFR3: vascular endothelial growth factor receptor 3
VEGF-A: vascular endothelial growth factor A
VEGF-B: vascular endothelial growth factor B
VEGF-C: vascular endothelial growth factor C
VEGF-D: vascular endothelial growth factor D
PlGF: placental growth factor
RMS: rhabdomyosarcoma
CDX: cell line-derived xenograft
PDX: patient-derived xenograft
DSRCT: desmoplastic small round cell tumor
