# Supplementary material for: Anti-VEGFR2 therapy delays growth of preclinical pediatric tumor models and enhances anti-tumor activity of chemotherapy
Source: Oncotarget. 2019 Sep 17;10(53):5523–33. doi: 10.18632/oncotarget.27148 (PMC6756863; doi:10.18632/oncotarget.27148)
Supplement: Supplementary file 1 [file oncotarget-10-5523-s001.pdf]

Anti-VEGFR2 therapy delays growth of preclinical pediatric tumor models and enhances anti-tumor activity of chemotherapy

SUPPLEMENTARY MATERIALS

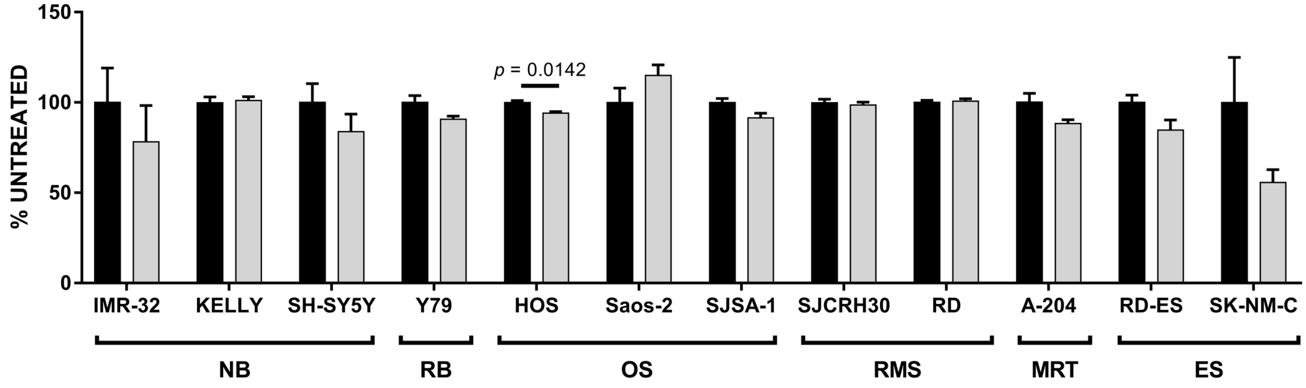

Supplementary Figure 1: Ramucirumab does not directly affect pediatric tumor cell viability. After the tumor-driven cord formation assays were completed, tumor cell health was measured by CellTiter Glo™. Results are presented normalized to the untreated control for each cell line. NB: neuroblastoma; RB: retinoblastoma; OS: osteosarcoma; RMS: rhabdomyosarcoma; MRT: malignant rhabdoid tumor; ES: Ewing's sarcoma. *p* value calculated by Student's t-test; error bars represent standard error of the mean (SEM).

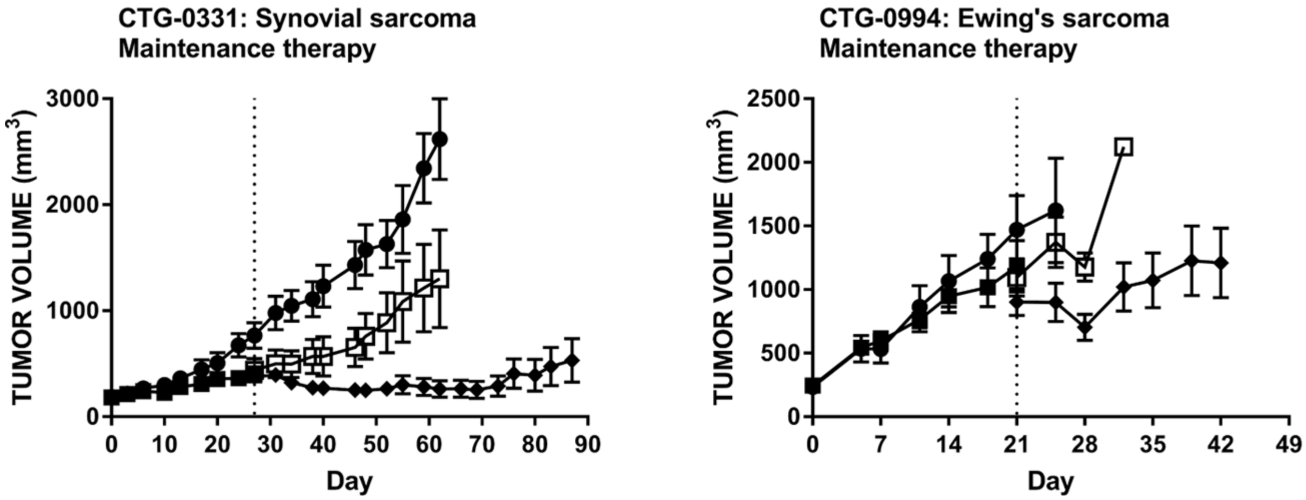

Supplementary Figure 2: DC101 is beneficial in a maintenance therapy setting in two preclinical models of pediatric sarcoma. Following treatment with doxorubicin (■; starting at Day 0), animals bearing either CTG-0331 synovial sarcoma (*left*) or CTG-0994 Ewing's sarcoma (*right*) were split into two groups at the timepoint indicated by the dotted line (Day 28 for CTG-0331 and Day 21 for CTG-0994). One received vehicle control (□) and the other received single agent DC101 (¨). Animals which only received vehicle throughout the study (●) are shown for reference.

Supplementary Table 1: *In vivo* models and experiment details

Model	Xenograft type	Tumor type	Mouse strain	Number of mice per arm				Maintenance therapy tested?
				Vehicle	DC101	Chemotherapy	Combination	
SJCRH30	cell line-derived	alveolar rhabdomyosarcoma	athymic nude	4	4	6	6	No
RD	cell line-derived	embryonal rhabdomyosarcoma	SCID	5	5	6	6	No
CTG-1213	patient-derived	embryonal rhabdomyosarcoma	athymic nude	5	5	10	5	Yes - no activity
CTG-0926	patient-derived	desmoplastic small round cell tumor	athymic nude	5	5	10	5	Yes - no activity
CTG-1458	patient-derived	desmoplastic small round cell tumor	athymic nude	5	5	5	5	No
RD-ES	cell line-derived	Ewing's sarcoma	SCID	6	6	6	6	No
CTG-0142	patient-derived	Ewing's sarcoma	athymic nude	5	5	10	5	Yes - no activity
CTG-0785	patient-derived	Ewing's sarcoma	athymic nude	5	5	10	5	Yes - no activity
CTG-0816	patient-derived	Ewing's sarcoma	athymic nude	5	5	10	5	Yes - no activity
CTG-0994	patient-derived	Ewing's sarcoma	athymic nude	5	5	10	5	Yes - see SFig 2
CTG-0143	patient-derived	Ewing's sarcoma	athymic nude	3	1	1	1	No
CTG-1651	patient-derived	Ewing's sarcoma	athymic nude	3	1	1	1	No
CTG-1663	patient-derived	Ewing's sarcoma	athymic nude	3	1	1	1	No
CTG-2003	patient-derived	Ewing's sarcoma	athymic nude	3	1	1	1	No
CTG-2113	patient-derived	Ewing's sarcoma	athymic nude	3	1	1	1	No
CTG-2174	patient-derived	Ewing's sarcoma	athymic nude	3	1	1	1	No
CTG-1072	patient-derived	hepatoblastoma	athymic nude	5	5	10	5	Yes - no activity
A-204	cell line-derived	malignant rhabdoid tumor	athymic nude	6	6	6	6	No
IMR-32	cell line-derived	neuroblastoma	SCID	6	6	6	6	No
KELLY	cell line-derived	neuroblastoma	SCID	5	5	5	5	No
SH-SY5Y	cell line-derived	neuroblastoma	athymic nude	5	5	5	5	No
CTG-0241	patient-derived	osteosarcoma	athymic nude	5	5	10	5	Yes - no activity
CTG-0242	patient-derived	osteosarcoma	athymic nude	5	5	10	5	Yes - no activity
CTG-0243	patient-derived	osteosarcoma	athymic nude	5	5	10	5	Yes - no activity
CTG-1064	patient-derived	osteosarcoma	athymic nude	5	5	10	5	Yes - no activity
Y79	cell line-derived	retinoblastoma	athymic nude	6	6	6	6	No
CTG-0331	patient-derived	synovial sarcoma	athymic nude	5	5	10	5	Yes - see SFig 2
CTG-1173	patient-derived	synovial sarcoma	athymic nude	5	5	10	5	Yes - no activity
CTG-1094	patient-derived	undifferentiated sarcoma	athymic nude	5	5	10	5	Yes - no activity

Italics denote models used for 'n of 1' studies.
